# Potential for Tight Junction Protein–Directed Drug Development Using Claudin Binders and Angubindin-1

**DOI:** 10.3390/ijms20164016

**Published:** 2019-08-17

**Authors:** Yosuke Hashimoto, Keisuke Tachibana, Susanne M. Krug, Jun Kunisawa, Michael Fromm, Masuo Kondoh

**Affiliations:** 1 Graduate School of Pharmaceutical Sciences, Osaka University, Osaka 565-0871, Japan; 2 Institute of Clinical Physiology, Charité–Universitätsmedizin Berlin, 12203 Berlin, Germany; 3 Laboratory of Vaccine Materials, Center for Vaccine and Adjuvant Research and Laboratory of Gut Environmental System, National Institutes of Biomedical Innovation, Health and Nutrition (NIBIOHN), Osaka 567-0085, Japan; 4 International Research and Development Center for Mucosal Vaccines, The Institute of Medical Sciences, The University of Tokyo, Tokyo 108-8639, Japan; 5 Department of Microbiology and Immunology, Kobe University Graduate School of Medicine, Kobe 650-0017, Japan; 6 Graduate School of Medicine and Graduate School of Dentistry, Osaka University, Osaka 565-0871, Japan

**Keywords:** tight junction, claudin, angulin, drug development, angubindin-1, *Clostridium perfringens* enterotoxin, *Clostridium perfringens* iota-toxin, antibody

## Abstract

The tight junction (TJ) is an intercellular sealing component found in epithelial and endothelial tissues that regulates the passage of solutes across the paracellular space. Research examining the biology of TJs has revealed that they are complex biochemical structures constructed from a range of proteins including claudins, occludin, tricellulin, angulins and junctional adhesion molecules. The transient disruption of the barrier function of TJs to open the paracellular space is one means of enhancing mucosal and transdermal drug absorption and to deliver drugs across the blood–brain barrier. However, the disruption of TJs can also open the paracellular space to harmful xenobiotics and pathogens. To address this issue, the strategies targeting TJ proteins have been developed to loosen TJs in a size- or tissue-dependent manner rather than to disrupt them. As several TJ proteins are overexpressed in malignant tumors and in the inflamed intestinal tract, and are present in cells and epithelia conjoined with the mucosa-associated lymphoid immune tissue, these TJ-protein-targeted strategies may also provide platforms for the development of novel therapies and vaccines. Here, this paper reviews two TJ-protein-targeted technologies, claudin binders and an angulin binder, and their applications in drug development.

## 1. Introduction

The boundaries between the inside of the body and the outside environment in the airway and gastrointestinal tract, and between the systemic circulation and tissues in the brain, eye, testis, and placenta, are separated by epithelial and endothelial cell sheets, respectively. The paracellular spaces between the adjacent cells in these sheets are sealed by a structural and functional component called the tight junction (TJ) [1]. TJs control the diffusion of ions, solutes, and water across the paracellular space to maintain homeostasis, and the loss of TJ integrity appears to be associated with the development of intestinal diseases [2,3], atopic dermatitis [4], and psychiatric disorders [5,6]. TJs also prevent mucosal and epidermal absorption of drugs and the delivery of drugs from the systemic circulation to the brain, eye, testis, and placenta.

A freeze-fracture replica electron microscopy analysis has shown that TJs consist of a meshwork of proteins called TJ strands [7]. In epithelial cells, these TJ strands are located at the apical side of the lateral membrane. TJs include various membrane proteins—including claudins, TJ-associated MARVEL proteins (occludin, tricellulin, and marvelD3), junctional adhesion molecules, and angulins—and these membrane proteins are anchored to intracellular scaffold proteins, e.g., of the zonula occludens protein family [8,9]. The physiological characteristics of TJs are determined by the specific combinations and mixing ratios of these TJ proteins [10,11,12]. TJ strands are dynamic structures that are repeatedly breaking and annealing, which transiently loosens the TJ seal and allows the stepwise diffusion of solutes across the meshwork and through the paracellular space [13].

There are two types of TJs: Bicellular, where two cells meet, and tricellular, where three cells meet. Bicellular TJ strands extend horizontally along the apical membrane but extend vertically when they reach a tricellular contact. Tricellular TJs seal the tubular structure created at tricellular contacts by the three vertically extending TJ strands and the three adjoining cell membranes [14].

A modulation of the structure of TJs to loosen the paracellular space can be used to increase mucosal and epidermal drug absorption, as well as drug delivery to the brain. Currently, sodium caprate and mannitol are used clinically to enhance paracellular drug absorption and drug delivery to the brain, respectively [15,16]. However, sodium caprate causes mucosal damage and lacks tissue-specificity [15,17]. The mannitol widened the interendothelial TJs to a radius of approximately 20 nm, followed by deliver chemicals, peptides, antibodies, and viral vectors to the brain [18]. Research into understanding the biochemical structure of TJs and the physiological roles of the various TJ proteins has provided insights that have been applied to the development of TJ protein–targeted drugs. Here, the efficacy and safety of claudin and angulin binders for the development of TJ-directed drugs is reviewed.

## 2. Claudins and Angulins

### 2.1. Claudins

**Figure 1:**
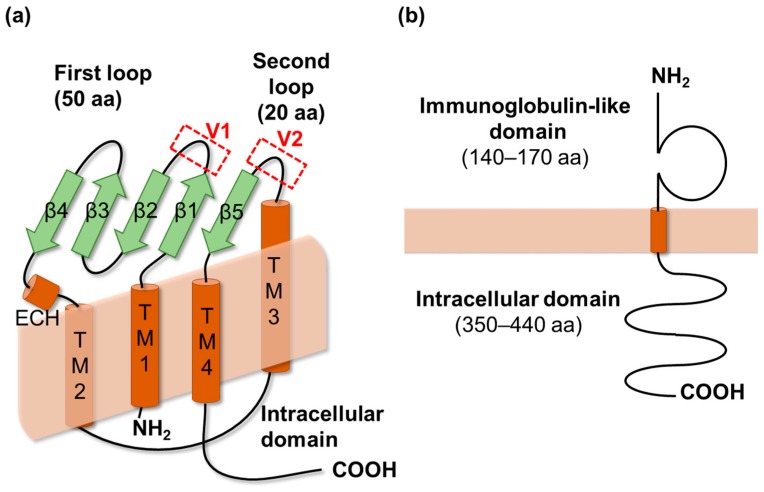
The representative structures of a claudin and an angulin. (**a**) Claudins are 20–27 kDa proteins containing four transmembrane (TM) domains and two extracellular loops. Extracellular loop 1 and 2 contains approximately 50 and 25 amino acids, respectively. TM domain 3 is much longer than the other three TM domains. Claudins also contain an extracellular helix (ECH) and two variable regions (V1 and V2). (**b**) Angulins are 60–70 kDa type I TM proteins containing an extracellular immunoglobulin-like domain and an intracellular tail. There is currently no structural information available for angulins. The secondary structural elements are shown as cylinders (α-helices) and arrows (β-strands). aa, amino acid.

Claudins were identified in 1998 as components of TJ strands that are crucial for the sealing of the intercellular space [19]. Currently, the mammalian claudin family comprises 27 proteins [20]. Since 2014, the crystal structures of these claudins have been gradually elucidated [21,22,23,24]. Claudins are tetra-transmembrane proteins containing two loops: The first contains four β-strands and an α-helix (extracellular helix), and the second contains a β-strand and the cell-surface-exposed transmembrane 3 domain (Figure 1a). Almost all claudins have the zonula occludens-1 binding motif at their C-terminal end. Claudins have *cis*-interactions within a cell membrane as well as *trans*-interactions with claudins in adjacent cell membranes. Both the *cis*- and *trans*-interactions are required for the building of TJ strands. The crystal structures of claudins have revealed that the *cis*-interaction between the extracellular helix and the transmembrane 3 domain leads to the formation of claudin strands within the cell membrane [21,22,23,24]. Claudins have two flexible loops, defined as variable regions: The first is between the β1- and β2-strands in the first extracellular domain, and the second is between the transmembrane 3 domain and the β5-strand in the second extracellular domain, with these flexible loops being involved in the formation of *trans*-interactions between claudin strands [25,26]. These flexible regions also appear to determine the characteristics of claudin-based TJs, because their homology is low among claudin members [21].

### 2.2. Angulins

The tricellular TJ is a specialized structure at the point of contact among three cells. Tricellulin, a member of the tight junction-associated MARVEL protein (TAMP) family, is an essential component of tricellular TJs [27]. Tricellulin does not form homophilic *trans*-interactions nor does it localize at tricellular contacts by itself [10,28]. Instead, it has to be recruited to tricellular contacts by angulins. Angulins are type I trans-membrane proteins with an extracellular immunoglobulin-like domain and a cytosolic tail (Figure 1b) [29]. There are three angulins: Angulin-1 (also known as lipolysis-stimulated lipoprotein receptor, LSR); angulin-2 (also known as immunoglobulin-like domain containing receptor 1, ILDR-1); and angulin-3 (also known as immunoglobulin-like domain containing receptor 2, ILDR-2). The interaction between phosphorylated Ser288 in the C-terminal tail of angulin-1 and the C-terminal cytosolic tail of tricellulin is responsible for the recruitment of tricellulin to the tricellular TJ [28,30]. Angulin-1 and -2 have a much greater ability to recruit tricellulin than angulin-3 [29]. The extracellular domain of angulin-2 may form a trimeric structure at the tricellular contact. However, the underlying mechanism remains unclear yet [31].

## 3. Claudin and Angulin Binders

Currently, most binders that target TJ proteins are either fragments of bacterial toxins (first-generation binders) or monoclonal antibodies (mAbs; second-generation binders) [32,33,34].

### 3.1. First-Generation Binders: Fragments of Bacterial Toxins

**Figure 2:**
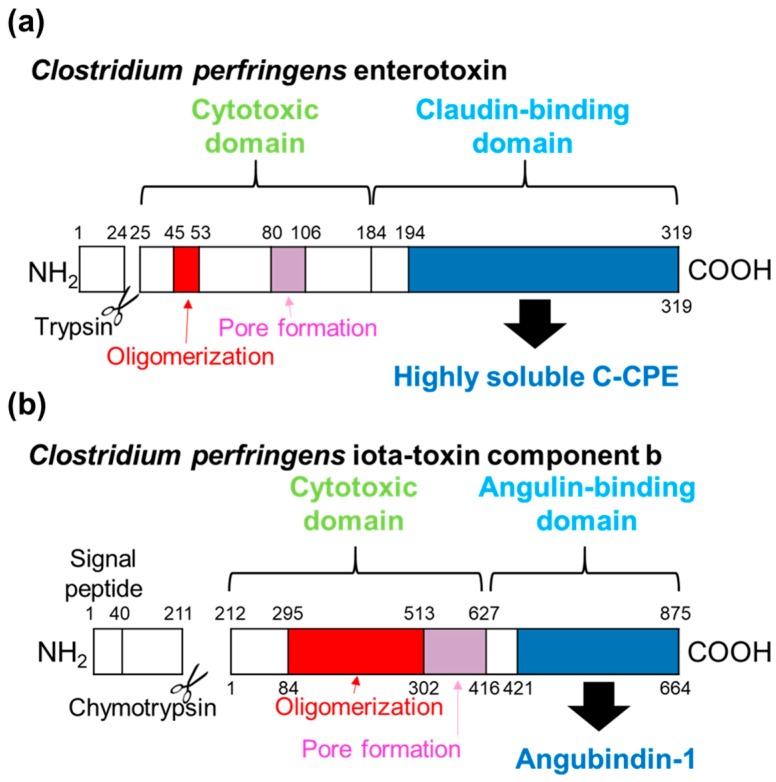
Schematic diagram showing the domains of first-generation claudin binders made from bacterial toxins. (**a**) *Clostridium perfringens* enterotoxin and (**b**) *C. perfringens* iota-toxin. Regions involved in oligomerization (red) and pore formation (pink) are indicated. Regions used as first-generation claudin binders are indicated in blue. The accession numbers for *C. perfringens* enterotoxin and iota-toxin are AOD41705 and CAA51960, respectively.

*Clostridium perfringens* enterotoxin (CPE) has two domains: The N-terminal cytotoxic domain, which is involved in oligomerization and pore formation, and the C-terminal receptor binding domain (C-CPE) [35] (Figure 2a). The CPE receptor (CPE-R) was identified, and CPE-R has significant similarity to the rat androgen withdrawal apoptosis protein (RVP1) in 1997 [36]. Two years after the identification of CPE-R and RVP1, claudin-3 and -4 have been identified to be RVP1 and CPE-R, respectively [37]. C-CPE binds to claudin-3 and -4 [38]. However, C-CPE also binds to claudin-6, -7, -8, -9, -14, and -19 [22,39,40]. The affinity of C-CPE to claudin-4 is approximately 0.5 nM [41]. The treatment of MDCK cells with C-CPE decreases TJ integrity [38]. The research into the generation of claudin binders using C-CPE as a template for site-directed mutagenesis has produced several C-CPE mutants: One broad-spectrum binder to claudin-1 to -5 and four relatively specific binders for claudin-3, -4, and -5 (Table 1).

*Clostridium perfringens* iota-toxin is a binary toxin composed of a cytotoxic domain (Ia) and a receptor-binding domain (Ib). Ib domain can be further classified into an Ia-binding domain (domain 1), oligomerization domain (domain 2), pore-formation domain (domain 3), and receptor-binding domain (domain 4). Domains 1 to 3 are involved in cytotoxicity [47]. Domain 4, which comprises amino acids 421–664, binds to the lipolysis-stimulated lipoprotein receptor without inducing cytotoxicity [48,49]. In 2013, the lipolysis-stimulated lipoprotein receptor was re-identified as angulin-1, the determining factor for the localization of tricellulin at tricellular TJs [28,29], and domain 4, the first tricellular TJ–specific modulator reported, was named angubindin-1 [50].

### 3.2. Second-Generation Binders: Monoclonal Antibodies

The TJ components are promising targets for the development of drugs to treat cancers and inflammatory bowel disease, for preventing infection by hepatitis C virus, and for the development of regenerative medicines. Antibodies are promising therapeutics for TJ-directed drug development because they bind to target proteins with high affinity and high specificity [51,52]. Thus, mAbs against the extracellular domains of the TJ components have been generated and their pharmaceutical activities are being investigated (Table 2).

## 4. Drug Delivery Using Claudin and Angulin Binders

The authors have recently completed a series of studies examining TJ binders that has provided new insights for TJ-directed drug development. Here, the proofs-of-concept for TJ-directed drug development using first- and second-generation claudin and angulin binders are introduced.

### 4.1. Mucosal Absorption

**Figure 3:**
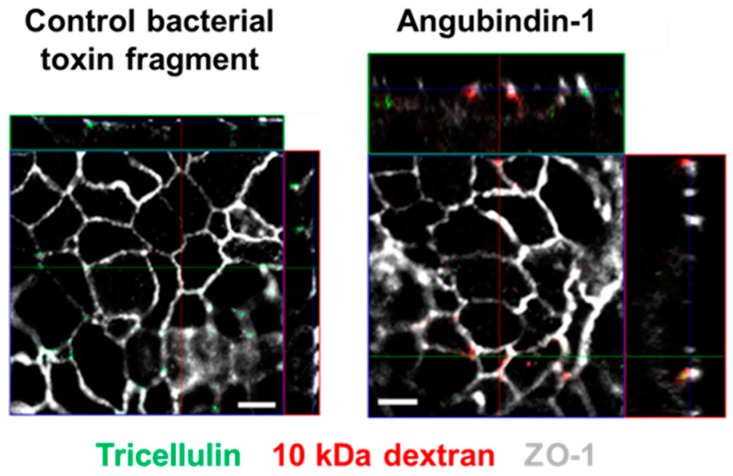
Visualization of the permeation of a macromolecule through tricellular TJs. HT-29/B6 cells were treated with control bacterial toxin fragment or angubindin-1 for 48 h. The cells were incubated with avidin and then with biotin-labeled tetramethylrhodamine-dextran 10 kDa (red) on the apical side of the insert for 1 h. The cells were then fixed and subjected to immunofluorescence analysis. The green signal represents tricellulin and the gray signal represents zonula occludens-1. Bars = 5 μm. The figure is reproduced from reference [50] with slight modifications and permission from the copyright holder.

Drug absorption across epithelia is either transcellular or paracellular [70]. One strategy for paracellular drug absorption is to loosen the TJs between adjacent epithelial cells. C-CPE increased jejunal absorption of dextran (4 kDa) 400-fold compared with sodium caprate, an absorption enhancer in current clinical use [17]. C-CPE also enhanced jejunal, nasal, and pulmonary absorption of a biologically active peptide [41]. The treatment of cells with angubindin-1 enhances the permeability of tricellular TJs to solutes up to 10 kDa (Figure 3). Angubindin-1 also enhanced jejunal absorption of dextran (4 kDa) [50]. These results demonstrate that modulation of bicellular and tricellular TJs could be useful strategy for the development of non-invasive drug-delivery systems.

### 4.2. Epidermal Absorption

The epidermis covers the outer body, preventing the passage of solutes and the absorption of drugs. However, epidermal administration is a potentially useful route of administration because it is noninvasive, can easily be stopped, and avoids first-pass metabolism [71]. The epidermal barrier comprises the stratum corneum and TJs in the stratum granulosum [72]. The analyses using knockout mice have revealed that claudin-1 is critical for TJ integrity in the stratum granulosum [73]. The treatment with an anti-claudin-1 mAb (7A5) weakened TJ integrity and enhanced the permeation of dextran (4 kDa) in an in vitro human epidermal model [53]. Thus, claudin-1-mediated modulation of the permeability of TJs in the stratum granulosum is a promising means of increasing epidermal drug absorption.

### 4.3. Cancer Targeting

Claudins are aberrantly expressed in many malignant tumors [74]. For example, in epithelium-derived tumors, TJ strands are disorganized and TJ proteins are distributed throughout the cell surface [75]. Claudin-3 and -4 are the most frequently overexpressed claudins in malignant tumors of prostate [76], breast [77], pancreatic [78], and ovarian cancers [79]. Claudin-1 and -2 are frequently overexpressed in colorectal cancers [80,81] and are associated with the promotion of tumor proliferation and invasiveness via the activation of intracellular signaling cascades [82,83,84].

**Figure 4:**
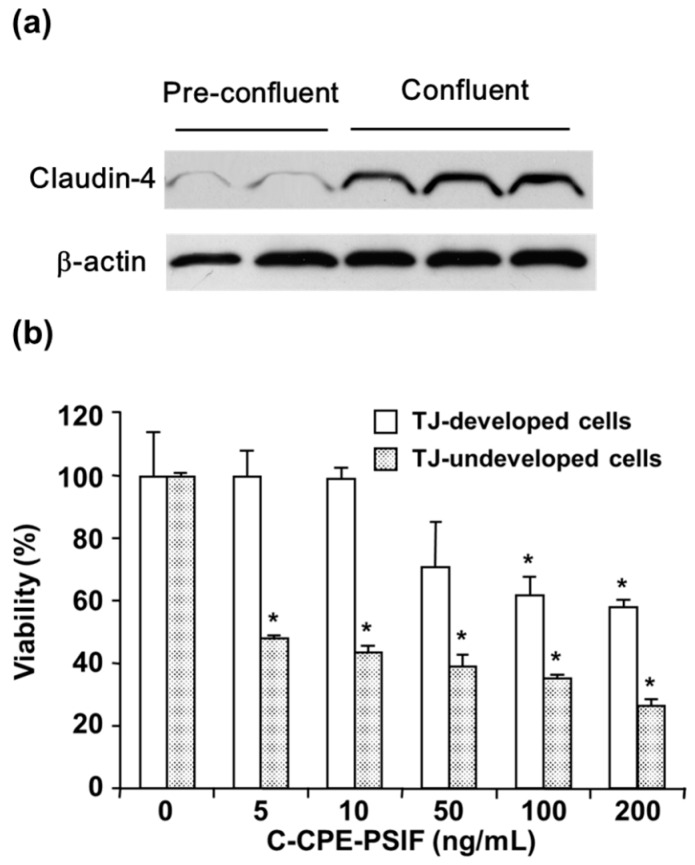
The effect of tight junction (TJ) maturity on the cytotoxicity of C-CPE fused with protein synthesis inhibitory factor (C-CPE-PSIF). Caco-2 cells were cultured to confluence or to preconfluence for 3 days to obtain cells with mature or immature TJs, respectively. (**a**) The cell lysates were subjected to western blotting. (**b**) The cells were treated with the indicated concentrations of C-CPE-PSIF for 48 h, and then cell viability was measured by WST-8 assay. The data are representative of at least three independent experiments. The data are shown as the mean ± S.D. (*n* = 3). * *p* < 0.05. The data are reproduced from reference [89] with slight modifications and permission from the copyright holder.

The various claudin-targeting molecules, including toxins, toxin fragments, and antibodies, have been generated for claudin-targeted cancer therapy [32,33,85]. For example, CPE has been used as an anti-cancer agent against pancreatic cancer overexpressing claudin-4 [86]. One issue with claudin-targeted therapies is that claudins are expressed not only in malignant tissues, but also in non-malignant tissues. However, most claudins in non-malignant tissues are localized within TJ complexes, whereas their localization is often dysregulated from TJ complexes to the cell surface in malignant tissues [87,88]. This study found that C-CPE fused with protein synthesis inhibitory factor (C-CPE-PSIF) may recognize claudins with aberrant localization, resulting in less binding and therefore, less toxicity to the normal cells [89]. Caco-2 is a human colon carcinoma cell line. Caco-2 cells form a polarized cell monolayer with well-developed TJs when confluent, and they are frequently used as a model of polarized normal epithelial cells. The claudin-4 protein level in the confluent culture (normal epithelial-like cells) was higher than in the subconfluent culture (carcinoma cells), and C-CPE-PSIF was cytotoxic to preconfluent (immature TJs) but not to postconfluent Caco-2 cells (mature TJs) (Figure 4) [89,90]. Similarly, anti-claudin-4 mAbs systemically administered to mice preferentially accumulated in tumor tissue rather than in normal tissue [67,91]. Together, these results suggest that claudins are potential targets for the development of cancer-targeted therapies.

### 4.4. Targeting Tissues Involved in Immunological Processes

Mucosal vaccination may be a useful immunization strategy because it is non-invasive and it activates both the mucosal and systemic immune responses. Epithelial cells associated with the mucosa-associated lymphoid tissues (MALT) include Peyer’s patches and nasopharynx and play pivotal roles in preventing the invasion of pathological microorganisms into the body by inducing the secretion of IgA [92]. MALT comprises various immune cells, including T cells, B cells, and dendritic cells, and is covered by follicle-associated epithelium. M cells are specialized epithelial cells in the follicle-associated epithelium that transport luminal antigens to immune cells in MALT by transcytosis [93]. In general, when antigen alone is orally or nasally administered, it fails to reach the MALT and so immune responses are not induced. Thus, the efficient delivery of antigen to MALT may provide effective mucosal vaccines.

**Figure 5:**
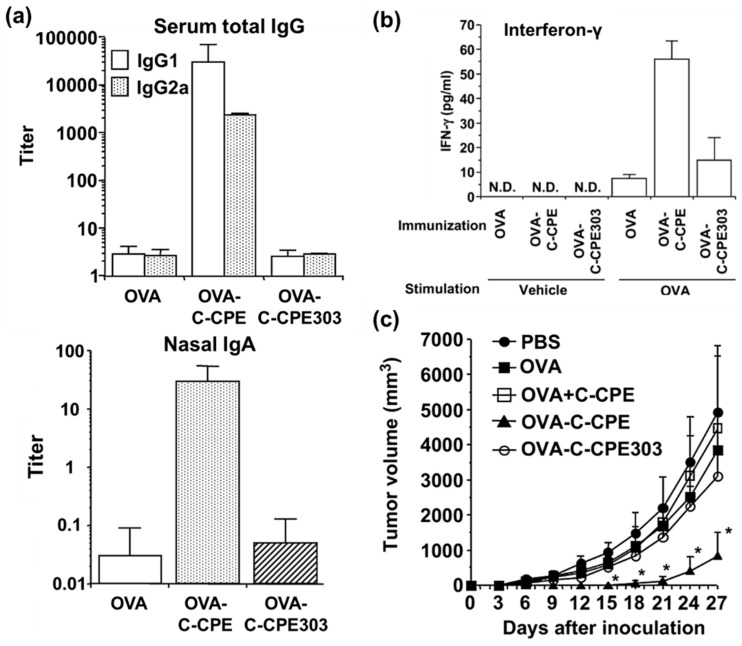
The effect of claudin-4-targeted mucosal immunization on systemic and mucosal immunity. (a and b) Mice were nasally immunized with ovalbumin (OVA), OVA-C-CPE, or OVA-C-CPE303 (which lacks the minimal claudin-binding region) (5 μg OVA in each formulation) once a week for 3 weeks. Seven days after the last immunization, serum and splenocytes were harvested. (**a**) The levels of serum IgG1 and IgG2a (upper panel) and nasal IgA (lower panel) were measured. (**b**) The splenocytes were stimulated with vehicle or OVA (1 mg/mL) for 24 h, and interferon-γ in the supernatant was measured. (**c**) Mice were nasally immunized with vehicle, OVA, a mixture of OVA and C-CPE, OVA-C-CPE, or OVA-C-CPE303 (5 μg OVA in each vaccine) once a week for 3 weeks. Seven days after the final immunization, the mice were injected subcutaneously with 1 × 10^6^ OVA-expressing EL4 (H-2b) cells. The tumor volumes were measured over time. The data are shown as the mean ± S.D. (*n* = 4). * *p* < 0.05. The data are reproduced from reference [97] with slight modifications and permission from the copyright holder.

Follicle-associated epithelium contains claudin-4-expressing cells, some of which are highly capable of capturing luminal antigen [94,95]. Claudin-4 is also expressed on the luminal surface of M cells [96]. Thus, claudin-4 targeting may be a promising strategy for delivering antigens to MALT. A nasally administered ovalbumin fused with C-CPE induced mucosal IgA production, systemic IgG production, and antigen-specific immune responses for preventing tumor growth (Figure 5) [97]. Of note, a simple mixture of C-CPE and antigen did not induce IgA production, indicating that the vaccination efficacy may be depending on the binding affinity of the C-CPE to claudins [97]. Nasal immunization with chimeric C-CPE-antigen did not induce mucosal injury [98]. The augmentation of the antigenicity of the first-generation binder C-CPE has been used to develop an adjuvant-free bivalent food poisoning vaccine [99]

### 4.5. Targeting Inflamed Tissues

Ulcerative colitis is a chronic, relapsing inflammatory bowel disease characterized by severe diarrhea and mucosal inflammation in the colon. The disruption of the colonic mucosal barrier leads to the activation of immune responses against bacterial and food fragments in the colon, followed by the development of ulcerative colitis [2,3]. Although claudin-2 is rarely expressed in normal colonic epithelial cells, its expression in the colon is upregulated in ulcerative colitis patients [80]. Inflammatory cytokines, including tumor necrosis factor-α (TNF-α), decrease the epithelial barrier integrity and upregulate the expression of claudin-2 [100]. Claudin-2 decreases the integrity of TJs by facilitating the formation of discontinuous TJ strands [101]. This suggests that the inhibition of claudin-2 may restore the disrupted mucosal barrier. Indeed, an anti-claudin-2 mAb (1A2) ameliorated TNF-α-induced reduction of TJ integrity in Caco-2 cells. Moreover, the co-treatment of the cells with anti-claudin-2 mAb and an anti-TNF-α mAb showed an additive effect on the restoration of the barrier [56].

### 4.6. Drug Deliavery to the Brain

Unlike peripheral capillaries, those in the brain lack fenestrations and have well-developed TJs that form the blood–brain barrier (BBB). More than 98% of small-molecular-weight drugs cannot pass the BBB [102]. Claudin-5 and angulin-1 are abundantly expressed by brain endothelial cells in mice [103]. Claudin-5- or angulin-1-knockout mice have a size-selectively loosened BBB [104,105]. These data suggest that claudin-5 and angulin-1 are candidate targets for opening the BBB. Indeed, a C-CPE mutant that can bind to claudin-5, angubindin-1, and anti-claudin-5 mAb was able to reduce the transepithelial/transendothelial electrical resistance (TER) in an in vitro model of the BBB [54,106]. Furthermore, in the mice, an angubindin-1-, but not claudin-5-binding C-CPE mutant increased the permeability of the BBB to allow passage of a 16-mer gapmer antisense oligonucleotide (5.3 kDa) [106]. No obvious adverse effects were observed in the mice in these experiments.

## 5. Safety of Claudin- and Angulin-Targeted Therapies

A series of proof-of-concept studies examining claudin and angulin targeting has provided insights into enhancing drug absorption, treating cancer and inflammatory diseases, improving vaccines, and obtaining drug delivery to the brain. No apparent adverse effects were observed in these studies. However, claudins and angulins play roles in the formation of the intercellular seal between and among epithelial cells and endothelial cells in many tissues. Therefore, ensuring the safety of claudin- and angulin-targeted drugs is critical for future drug development.

The knockout and knockdown analyses of the genes encoding claudins and angulins have shown that there are risks associated with claudin- and angulin-targeted therapeutics (Table 3). For instance, the inhibitors of claudin-1 and -5 may induce atopic dermatitis and schizophrenia-like symptoms via the inhibition of the epidermal barrier and the BBB, respectively [4,6]. Claudin-2, -4, and angulin-2-targeted drugs may induce renal impairment with respect to the reabsorption of ions and water [107,108,109]. A deletion in exon 1 of claudin-1 results in neonatal ichthyosis and sclerosing cholangitis syndrome in humans [110]. A deletion of 1.5 to 3.0 Mb of human chromosome 22q11.2 that includes the *claudin-5* gene is associated with the development of schizophrenia [111]. A single nucleotide polymorphism in *claudin-5* is also associated with the development of schizophrenia [112,113].

The expression profiles of claudins and angulins differ among tissues (Table 4). The specific claudin ratio is critical for the functions of TJs [115]. Thus, the toxicity of claudin-directed drugs should be carefully investigated, especially if the target claudins are also expressed in non-target tissues. Claudins and angulins in TJs are embedded in the lateral cell membranes and extend into the intercellular space. The TJ cavity is estimated to be 0.5 nm under physiological conditions [116,117]. Large binders, such as antibodies, which are unable to access proteins embedded in TJs, are a promising modality for treating cancers and for improving the effectiveness of vaccines because in these conditions the target claudins are exposed on the cell surfaces [34,87,88,96]. TJ components are potent targets for the development of many novel therapies, but targets and drug modalities must be optimized to afford an acceptable risk–benefit balance.

## 6. Conclusions

TJ binders are classified as first-generation binders (toxins and their fragments), and second-generation binders (antibodies) [32,33,85]. The augmentation of the antigenicity of the first-generation binder, C-CPE, has been used to develop an adjuvant-free vaccine [97,99]. Second-generation binders are being used to develop cancer therapies (Table 2). For example, an anti-claudin-18.2 mAb is undergoing clinical study for the use in the treatment of gastric (phase III study) and pancreatic cancer (phase II study) [NCT03504397; NCT03816163].

The other application of TJ binders is to enhance the mucosal and epidermal absorption of drugs and to deliver drugs to the brain by modulating the permeability of TJs. The currently available TJ binders are toxin fragments and antibodies. However, the generation of novel TJ binders, such as peptides and chemicals, is now needed because of the potential antigenicity of toxins and the costs associated with antibody preparation. A high-throughput screening system for claudin-4 binders based on the time resolved fluorescence resonance energy transfer in a chemical library was developed [126]. In the future, the generations of peptide- and chemical-type of binders are expected to accelerate.

## Figures and Tables

**Table 1:** List of claudin-binding mutants of C-terminal receptor binding domain of *Clostridium perfringens* enterotoxin.

Binder Type	Mutated Regions	Ref.
Negative-binding mutant	Y306A/L315A	[42]
Broad-spectrum binder (m19)	S304A/S305P/S307R/N309H/S313H	[43]
Enhancing specificity to claudin-3	L223A/D225A/R227A	[44]
Enhancing specificity to claudin-4	L254A/S256A/I258A/D284A	[44]
Enhancing specificity to claudin-5	Y306W/S313H	[45]
Improved specificity to claudin-5	N218Q/Y306W/S313H	[46]

**Table 2:** List of current monoclonal antibodies against tight junction components.

Indicated Application or Disease	Target	Monoclonal Antibody Name	Ref. or ClinicalTrials.gov Identifier
Modulation of epidermal barrier	Claudin-1	7A5	[53]
Modulation of blood–brain barrier	Claudin-5	R9, R2, 2B12	[54,55]
Inflammatory bowel disease	Claudin-2	1A2	[56]
Hepatitis C virus infection	Claudin-1	OM-7D3-B3, 3A2	[57,58]
	Occludin	1-3, 67-2	[59,60]
Gastric cancer (phase III study)	Claudin-18.2	IMAB362	NCT03504397
Pancreatic cancer (phase II study)	Claudin-18.2	IMAB362	NCT03816163
Germ cell tumor (phase II study)	Claudin-6	IMAB027	NCT03760081
Cancers (phase I or pre-clinical study)	Claudin-1	3A2, 6F6	[61,62]
	Claudin-2	1A2	[63]
	Claudin-3	KMK3953, IgGH6	[64,65]
	Claudin-4	KM3934, 5D12	[66,67]
	Angulin-1	#1-25	[68]
	Angulin-3	BAY1905254	NCT03666273
Regenerative medicine	Claudin-6	clone 342927	[69]

**Table 3:** Phenotypes of representative claudin- or angulin-knockout or -knockdown mice.

	Phenotype of Knockout (KO) or Knockdown (KD) Mice	Ref.
Claudin-1	Atopic dermatitis (KD)	[4]
Claudin-2	Impaired renal Na^+^, Cl^−^, and water reabsorption (KO)	[108]
Claudin-3	Increased hepatocyte permeability to phosphate ion (KO)	[114]
Claudin-4	Impaired renal Ca^2+^ and Cl^−^ reabsorption (KO)	[107]
Claudin-5	Schizophrenia-like symptoms (KD)	[6]
Angulin-2	Impaired renal water reabsorption and colonic water absorption (KO)	[109]

**Table 4:** Claudin and angulin expression in representative tissues in a mouse or rat.

	Claudin						Angulin			Ref.
	1	2	3	4	5		1	2	3	
Epidermal cells (stratum granulosum)	+	-	-	+	-		+	-	-	[29,73]
Nasal epithelial cells	+	-	+	+	+		+	+	-	[29,118]
Lung (alveoli)	+	-	+	+	+		+	-	-	[29,119]
Small intestine (jejunum)	+	+	+	-	+		+	-	-	[29,109,120]
Colon (surface)	+	-	+	+	+		-	+	-	[29,109,120]
Liver	+	+	+	-	-		+	-	-	[29,121,122]
Kidney (glomerulus)	+	+	-	-	-		-	+	-	[29,123]
Kidney (proximal tube)	-	+	-	-	-		+	-	-	[29,123]
Kidney (thin ascending limb of the loop of Henle)	-	-	+	+	-		-	+	-	[29,123]
Kidney (collecting duct)	-	-	+	+	-		-	+	-	[29,109,123]
Brain endothelial cells	-	-	-	-	+		+	-	+	[103,105]
Brain ependymal cells	+	+	+	-	-		-	-	+	[29,124]
Lung endothelial cells	-	-	-	-	+		-	-	-	[125]

+, expressed; -, not detected.

## Abbreviations

BBB	Blood–brain barrier
CPE	Clostridium perfringens enterotoxin
CPE-R	CPE receptor
C-CPE	C-terminal domain of CPE
C-CPE-PSIF	C-CPE fused with protein synthesis inhibitory factor
ECH	Extracellular helix
KD	Knockdown
KO	Knockout
mAb	Monoclonal antibody
MALT	Mucosa-associated lymphoid tissues
RVP1	Rat androgen withdrawal apoptosis protein
TER	Transepithelial/transendothelial electrical resistance
TJ	Tight junction
TM	Transmembrane
TNF-α	Tumor necrosis factor-α
